# Polarimetric Sensitivity to Torsion in Spun Highly Birefringent Fibers

**DOI:** 10.3390/s19071639

**Published:** 2019-04-05

**Authors:** Dominik Kowal, Gabriela Statkiewicz-Barabach, Marta Bernas, Maciej Napiorkowski, Mariusz Makara, Lidia Czyzewska, Pawel Mergo, Waclaw Urbanczyk

**Affiliations:** 1 Department of Optics and Photonics, Faculty of Fundamental Problems of Technology, Wroclaw University of Science and Technology, Wybrzeze Wyspianskiego 27, 50-370 Wroclaw, Poland; dominik.kowal@pwr.edu.pl (D.K.); marta.bernas@pwr.edu.pl (M.B.); maciej.napiorkowski@pwr.edu.pl (M.N.); waclaw.urbanczyk@pwr.edu.pl (W.U.); 2 Laboratory of Optical Fiber Technology, Maria Curie-Sklodowska University, pl. M. Curie-Sklodowskiej 3, 20-031 Lublin, Poland; m.makara@poczta.umcs.lublin.pl (M.M.); lidia.czyzewska@poczta.umcs.lublin.pl (L.C.); pawel.mergo@poczta.umcs.lublin.pl (P.M.)

**Keywords:** spun fibers, highly birefringent fibers, sensitivity to torsion

## Abstract

We report on experimental studies of polarimetric sensitivity to torsion in spun highly birefringent fibers. Two classes of spun fibers were examined, namely spun side-hole fibers and birefringent microstructured fibers with different birefringence dispersion, spin pitches, and spin directions. The polarimetric sensitivity to torsion was determined by monitoring a displacement of the spectral interference fringes arising in the output signal because of interference of polarization modes and induced by an additional fiber twist. Both the experimental results and the analytical predictions showed that the sensitivity to torsion normalized to the fringe width in the spun highly birefringent fibers increased asymptotically with the twist rate to the value of 1/ π rad^−1^. We have also studied the polarimetric response to temperature in the spun side-hole fibers. We have found that, in contrast to the torsional sensitivity, the temperature sensitivity decays asymptotically to zero with increasing fiber twist rate. Therefore, the spun fibers with short spin pitches are especially well suited for torsion measurements because the torsional sensitivity and the range of linear response are both enhanced in such fibers, while at the same time, the cross-sensitivity to temperature is reduced.

## 1. Introduction

The first spun fibers [1,2] were reported in the early 1980s, but their applications were initially limited because of difficulties with fabrication of short spin pitches and the lack of effective modelling methods. These problems have been successfully addressed in the last two decades [3,4,5,6,7], which boosted the interest in this new class of fibers, studied currently for new wave phenomena, as well as novel applications. One of the most prominent properties of the spun fibers is their ability to support optical modes with predefined orbital angular momentum (OAM) [5,8,9], which allowed for the development of novel spatial division multiplexing methods for optical transmission based on OAMs’ discrimination. Moreover, the spun fibers are also applied for increasing the performance of single-mode lasers [10,11], reducing polarization mode dispersion [12], and for sensing purposes. From their first appearance, the spun fibers were investigated for use in magnetic field and electric current sensing [13]. Recently, a new phenomenon has been observed in spun conventional and microstructured fibers, which is the resonant coupling between the core and cladding modes arising for appropriate combinations of fiber spin pitch and wavelength. This has resulted in the development of the so-called chiral fiber gratings [3,4,14,15,16,17,18], which can be used for sensing temperature, elongation, liquid level, or fiber twist [19,20,21] by monitoring the spectral shift of the resonance loss peak.

Various fiber-optic sensors for measurement of torsion have been already reported in the literature. Possible approaches include the use of Sagnac interferometers [22,23,24], Mach-Zehnder interferometers [25,26], tilted fiber Bragg gratings [27,28], or long-period fiber gratings [29,30]. Since the first observation of resonant coupling between the core and cladding modes in chiral fiber gratings [3], such structures have been also studied for torsion sensing, either in a single- [31] or double-grating configuration [19]. It was also shown in [32] that the sensitivity of chiral fiber gratings to torsion may be tuned by varying the spin pitch of the grating. The influence of the twist on the characteristics of fibers with high linear birefringence was also investigated in [1,33].

In this paper, we study the effect of torsion-induced birefringence variation in spun highly birefringent fibers (SHBFs), which can be potentially used for measuring directional torsion. The SHBFs are typically fabricated by twisting the fibers (during the drawing process or afterward) with initial linear birefringence induced by the ellipticity of the core [34], stress-applying elements [35], or microstructured cladding asymmetry [36]. The SHBFs support helical elliptically-polarized modes with azimuths following the fiber symmetry axes, opposite handedness, and the ellipticity angle defined by the ratio of the linear beat length and spin pitch. When such a fiber is placed between two linear polarizers, an interference pattern is observed in the spectrum of the transmitted light. Twisting the SHBF results in the variation of the phase delay between elliptically-polarized modes, which is manifested in the shift of the spectral interference fringes. The effect of varying the phase delay between the polarization modes in the SHBF under elastic twist was studied in [37]. The periodic twist-induced changes of the output intensity measured for a single wavelength were used to calculate the linear beat length of the SHBF. In this work, we investigate the effect of twist-induced shift of the interference spectrum at the output of the SHBF as a possible way for measuring the elastic twist itself. A similar approach for measuring twist using the hollow-core photonic bandgap fiber with two linear in-fiber polarizers fabricated by a CO_2_ laser beam has been reported in [38]. Moreover, in [39], the variation of transmitted power was examined while twisting the birefringent microstructured fiber placed between the polarization controller and output linear polarizer. It should be emphasized that the fibers used in [38,39] as the twist sensors were not spun initially, which according to our findings, is crucial for the sensor performance. 

In this work, we examine and compare the sensitivity to torsion for two families of SHBFs with different birefringence dispersions, namely spun side-hole fibers (SHFs) and birefringent microstructured fibers (BMFs) with a pair of large holes. To the best of our knowledge, these are the first measurements of the polarimetric sensitivity of the SHBFs to torsion. Our experimental results show that the polarimetric sensitivity to torsion of the SHBFs and the range of linear response are greater in fibers with shorter spin pitches. Simultaneously, we show that the polarimetric sensitivity to temperature decreases against fiber initial twist rate. To support our experimental findings, we have derived analytical formulas linking the torsion-induced shift of the interference spectrum with the spin pitch and built-in linear birefringence of the SHBFs. The measured values of the torsional and temperature sensitivities for different fibers show good agreement with our analytical predictions.

## 2. Experimental Setup and Sensitivity Analysis

**Figure 1:**
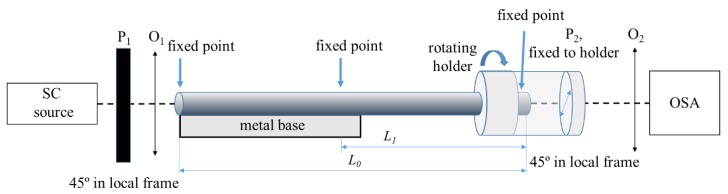
Schematic view of the setup for measuring the response of spun highly birefringent fibers to torsion. SC—supercontinuum source, OSA—optical spectrum analyzer.

An experimental setup for measuring the sensitivity of the SHBFs to torsion is schematically shown in Figure 1. A broadband light emitted from the supercontinuum source (SC) was linearly polarized at 45° in reference to the symmetry axes of the SHBF (local frame) by the dichroic linear polarizer P_1_ (operation range from 0.6–1.8 μm) and coupled to the fiber through the microscopic objective O_1_. The input end of the SHBF of total length *L*_0_ was fixed to a stiff metal base, and its angular orientation remained constant during the measurements. The output section of the fiber of length *L*_1_, whose end was fixed together with the polarizer P_2_ in the rotatable holder, was subjected to twist. The distance between fixed point of the fiber and the twisted end was equal to *L_1_ =* 25 cm. The second linear polarizer P_2_ at the fiber output was set at 45° in reference to the symmetry axes of the fiber to obtain the maximum contrast of the interference fringes in the output spectrum. The polarizer P_2_ was then rotated together with the output end of the fiber; therefore, the relative orientation of the fiber symmetry axes and the polarizer P_2_ remained constant during the experiments. This allows using the local frame in the analysis of torsional sensitivity presented in the following paragraphs.

The difference in the effective indices of the elliptically-polarized modes propagating in the SHBFs is called elliptical birefringence Δ*n_e_*. In the local frame used in our experiments (i.e., rotating with the fiber symmetry axes), the elliptical birefringence is related to built-in linear birefringence Δ*n_l_* and twist-induced circular birefringence Δ*n_c_* in the following way [40]:

(1) $$\Delta n_{e}=\sqrt{{\left(\Delta n_{l}\right)}^{2}+{\left(\Delta n_{c}\right)}^{2}},$$

where the circular birefringence resulting from the fiber initial inelastic twist is expressed by [40]:

(2) $$\Delta n_{c}=\frac{\lambda }{\pi L_{0}}\left|\alpha \right|.$$

In the above equation, *L_0_* indicates the fiber length, *α* the initial twist angle, and *λ* the light wavelength. The positive sign of *α* is assigned for a clockwise fiber twist. The group elliptical birefringence does not depend on the choice of the reference system and is expressed as:

(3) $$\Delta N_{e}=\Delta n_{e}-\lambda \frac{d\Delta n_{e}}{d\lambda }.$$

A similar relation holds for the built-in linear group birefringence Δ*N_l_*. It has been recently shown [41] that these two parameters are related in the following way:

(4) $$\Delta N_{e}\left(\lambda \right)=\frac{\Delta n_{l}}{\sqrt{\Delta n_{l}^{2}+\Delta n_{c}^{2}}}\Delta N_{l}\left(\lambda \right).$$

In the experiments conducted in the setup shown in Figure 1, we have monitored the displacement of the interference fringes induced by an additional elastic twist Δ*α* applied to the end-section of the fiber of length *L_1_*. Because of circular birefringence induced by shear strain, the fiber elastic torsion by an angle Δ*α* induces the rotation of the polarization state at the fiber output by Δ*α*(1 + 1/2*μ*), where *μ =* 6.85 has been experimentally determined for silica fibers [1,42]. In the following analysis, we disregard the term 1/2*μ* representing the effect of shear strain. Physically, this means that we do not distinguish between nonelastic (spun) and elastic (torsion) twist of the fiber. Such an assumption significantly simplifies the derived analytical formulas at the expense of relatively small inaccuracy of the order of 7%. 

The interference minimum of the order *q* arises at the wavelength satisfying the following condition:

(5) $$\Delta \varphi \left(\lambda ,\alpha \right)=\frac{2\pi \Delta n_{e}(\lambda ,\alpha )L_{0}}{\lambda }=\;(2q+1)\pi ,$$

where Δ*φ* is the phase delay between elliptically-polarized eigenmodes. Twisting the fiber end-section by an angle *dα* affects the phase delay Δ*φ*, which in turn causes the shift of the observed fringe by *dλ*. The two parameters are related to each other in the following way:

(6) $$\frac{\partial \Delta \varphi \left(\lambda ,\alpha \right)}{\partial \lambda }d\lambda =-\frac{\partial \Delta \varphi \left(\lambda ,\alpha \right)}{\partial \alpha }d\alpha \;.$$

The ratio of the twist-induced spectral shift of the interference fringe *dλ* to the twist angle *dα* defines the fiber sensitivity to torsion $K_{\alpha }^{\prime }$ expressed in nm/rad:

(7) $$K_{\alpha }^{\prime }=\frac{d\lambda }{d\alpha }.$$

The derivatives in Equation (6) are expressed as follows:

(8) $$\frac{\partial \Delta \varphi \left(\lambda ,\alpha \right)}{\partial \lambda }=-\frac{2\pi L_{0}\Delta N_{e}}{\lambda^{2}},$$

and:

(9) $$\begin{array}{c} \frac{\partial \Delta \varphi \left(\lambda ,\alpha \right)}{\partial \alpha }=\frac{2\pi L_{1}}{\lambda }\frac{\partial \Delta n_{e}}{\partial \alpha }=\\ \frac{2\pi L_{1}}{\lambda }\left[\frac{\Delta n_{c}}{\sqrt{{\left(\Delta n_{c}\right)}^{2}\;+{\left(\Delta n_{l}\right)}^{2}}}\right]\frac{\partial \Delta n_{c}}{\partial \alpha }=\pm 2\left[\frac{\Delta n_{c}}{\sqrt{{\left(\Delta n_{c}\right)}^{2}\;+{\left(\Delta n_{l}\right)}^{2}}}\right]. \end{array}$$

Using the above relations, we can finally obtain the formula for the torsional sensitivity of the SHBF:

(10) $$K_{\alpha }^{\prime }=\frac{d\lambda }{d\alpha }\;=\pm \frac{\lambda^{2}}{\pi L_{0}\Delta N_{e}}\left[\frac{\Delta n_{c}}{\sqrt{{\left(\Delta n_{c}\right)}^{2}\;+{\left(\Delta n_{l}\right)}^{2}}}\;\right],$$

which can be further simplified using Equation (4):

(11) $$K_{\alpha }^{\prime }=\frac{d\lambda }{d\alpha }\;=\pm \frac{\lambda^{2}}{\pi L_{0}\Delta N_{l}}\;\frac{\Delta n_{c}}{\Delta n_{l}}.$$

One should note that the positive sign in the above expression applies for the fibers with a clockwise initial twist. In order to obtain the expression for torsional sensitivity that is independent of the fiber length *L_0_*, we normalize it to the spectral width Δ*λ_p_* of the interference fringe (i.e., the distance between the adjacent extrema of the interference pattern) given by:

(12) $$\Delta \lambda_{p}=\frac{\lambda^{2}}{L_{0}\Delta N_{e}}.$$

The normalized torsional sensitivity takes the form:

(13) $$\begin{array}{c} K_{\alpha }=\frac{K_{\alpha }^{\prime }}{\Delta \lambda_{p}}\;=\pm \frac{\lambda^{2}}{\pi L_{0}\Delta \lambda_{p}\Delta N_{l}}\;\frac{\Delta n_{c}}{\Delta n_{l}}=\\ \pm \frac{\Delta N_{e}}{\pi \Delta N_{l}}\;\frac{\Delta n_{c}}{\Delta n_{l}}=\pm \frac{1}{\pi \sqrt{1\;+{\left(\Delta n_{l}/\Delta n_{c}\right)}^{2}}}, \end{array}$$

where Equation (4) was used again to eliminate the ratio Δ*N_e_* /Δ*N_l_*. The above formula can be also expressed in terms of linear beat length *L_L_* and spin pitch *L_T_*, which are related to linear and circular birefringence in the following way:

(14) $$L_{L}=\frac{\lambda }{\Delta n_{l}}\;$$

and respectively:

(15) $$L_{T}=\frac{2\lambda }{\Delta n_{c}}\;$$

where the factor of two is related to the fact that rotation angle of the polarization plane is twice smaller than the phase shift between circularly-polarized modes. 

This finally yields:

(16) $$K_{\alpha }=\pm \frac{1}{\pi \sqrt{1\;+{\left(L_{T}/2L_{L}\right)}^{2}}}.$$

**Figure 2:**
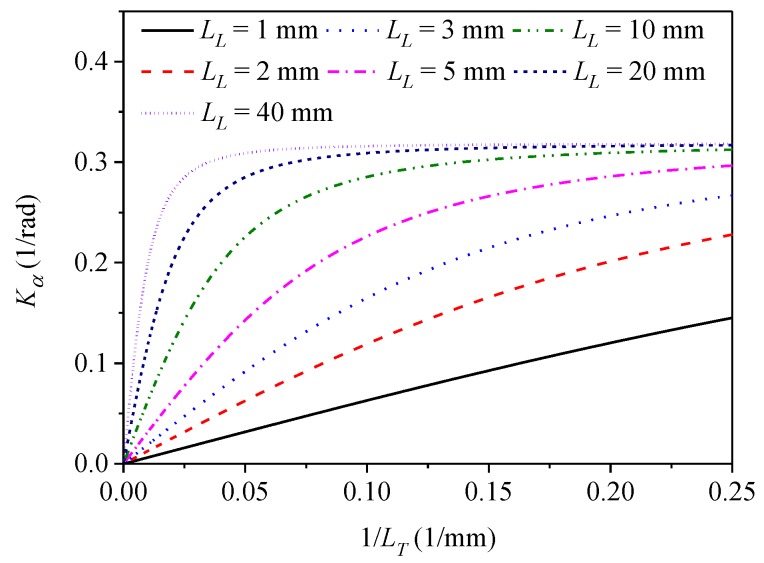
Theoretical curves *K_α_* plotted against inverse of the fiber spin pitch *1/L_T_* for different values of the linear beat length *L_L_*.

The derived formula shows that for large values of circular birefringence Δ*n_c_* ≫ Δ*n_l_ (L_T_* ≪ *L_L_)* the normalized torsional sensitivity *K_α_* tends asymptotically to a constant value equal to 1/π rad^−1^. For small values of circular birefringence Δ*n_c_* ≪ Δ*n_l_ (L_T_* ≫ *L_L_)*, the normalized sensitivity may be expressed as follows:

(17) $$K_{\alpha }=\;\pm \frac{\Delta n_{c}}{\pi \sqrt{{\left(\Delta n_{c}\right)}^{2}\;+{\left(\Delta n_{l}\right)}^{2}}}\approx \pm \frac{\Delta n_{c}}{\pi \Delta n_{l}}=\pm \frac{2L_{L}}{\pi L_{T}},$$

which implies a linear growth of *K_α_* with Δ*n_c_* or 1*/L_T_*. The theoretical value of the normalized sensitivity *K_α_* is plotted in Figure 2 against inverse of spin pitch 1*/L_T_* for several values of the linear beat length *L_L_*. 

Using a similar approach, one can evaluate the effect of initial twist on polarimetric sensitivity to temperature (normalized to fringe width) in spun highly birefringent fibers, which is defined as follows:

(18) $$K_{T}=\frac{1}{\Delta \lambda_{p}}\frac{d\lambda }{dT}.$$

**Figure 3:**
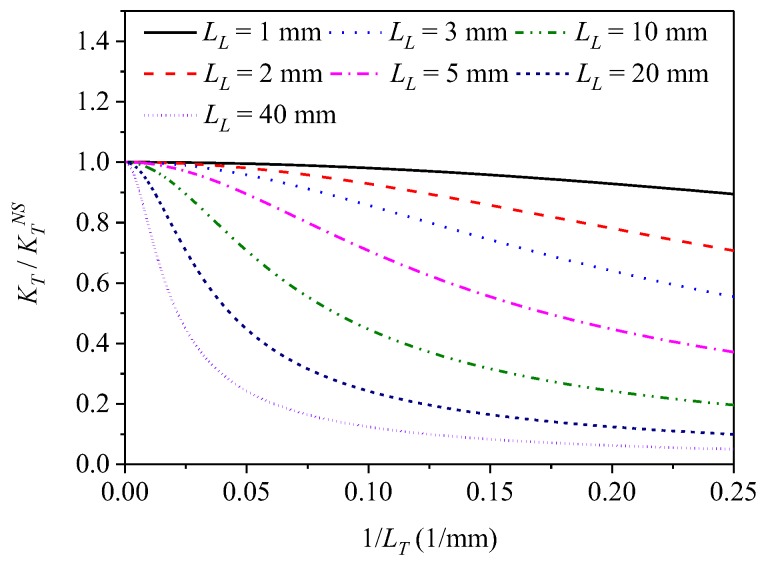
Theoretical curves *K_T_/K_T_^NS^* plotted against the inverse of the fiber spin pitch *1/L_T_* for different values of the linear beat length *L_L_*.

In the temperature sensitivity analysis, we have disregarded the effect of temperature-induced fiber elongation because of the very small value of the temperature expansion coefficient for silica glass, *η* = 5.5 × 10^−7^ K^−1^. As explained in [43], the fiber polarimetric sensitivity to temperature is a sum of two factors, i.e., the temperature-induced variation of modal birefringence represented by the term *∂*Δ*n/∂T* and the temperature-induced fiber elongation represented by the term *η*Δ*n*. The primary physical phenomena behind the polarimetric sensitivity to temperature depends on fiber construction. In fibers with stress-applying elements, the sensitivity *K_T_* is mostly related to the release of thermal stress caused by increasing temperature. In fibers with elliptical cores, *K_T_* mostly depends on the difference in the thermo-optic coefficient *dn/dT* between the core and the cladding, while in pure silica, microstructured fibers on the index difference increase between air holes and silica glass and on the temperature-induced increase of the lattice pitch. Even in the case of microstructured fibers showing very small sensitivity to temperature, the contribution to *K_T_* related to longitudinal fiber expansion is negligible [43]. Therefore, it is fully justified to disregard the effect of temperature-induced fiber elongation in Equation (9), which after doing rearrangements similar to the case of the torsional sensitivity, leads to the following expression for *K_T_*:

(19) $$K_{T}=\frac{L_{1}}{\lambda }\frac{\partial \Delta n_{e}}{\partial T}=\frac{L_{1}}{\lambda }\frac{\partial \Delta n_{l}}{\partial T}\frac{\Delta n_{l}}{\sqrt{{\left(\Delta n_{c}\right)}^{2}\;+{\left(\Delta n_{l}\right)}^{2}}}$$

and consequently:

(20) $$K_{T}=K_{T}^{NS}\frac{\Delta n_{l}}{\sqrt{{\left(\Delta n_{c}\right)}^{2}\;+{\left(\Delta n_{l}\right)}^{2}}},$$

where *L_1_* indicates the length of the fiber section exposed to temperature variation and $K_{T}^{NS}$ is the normalized temperature sensitivity of non-spun fiber with linear birefringence Δ*n_l_*. The above relation shows that the sensitivity to temperature is the highest for non-spun fiber and asymptotically tends to zero for increasing fiber twist rate. In Figure 3, the ratio $K_{T}^{}/K_{T}^{NS}$ is shown as a function of the inverse of the fiber spin pitch 1/*L_T_*. We therefore conclude that application of spun highly birefringent fibers with short spin pitches to twist measurement are particularly beneficial, as the high twist rate increases the normalized sensitivity to torsion and simultaneously decreases the normalized sensitivity to temperature, thus reducing the cross-sensitivity effect. 

It is worth mentioning that in many sensing applications of highly birefringent fibers reported in the literature [44], the polarimetric sensitivity to temperature is expressed in rad/K × m and denotes additional phase shift between polarization modes induced by a temperature increase of 1 K in a 1 m-long fiber:

(21) $$S_{T}=\frac{1}{L_{1}}\frac{d\Delta \varphi }{dT}=\frac{2\pi }{\lambda }\frac{\partial \Delta n_{e}}{\partial T}\;,$$

where in the above relation, similarly to Equation (19), the term $2\pi \eta \Delta n_{e}/\lambda$ related to temperature-induced fiber elongation is disregarded. The two temperature sensitivities are therefore related in the following way:

(22) $$K_{T}=\frac{L_{1}}{2\pi }S_{T},$$

which leads to the conclusion that also the ration $S_{T}^{}/S_{T}^{NS}$ decays asymptotically to zero with increasing fiber twist rate according to Equation (20).

## 3. Experimental Results

**Figure 4:**
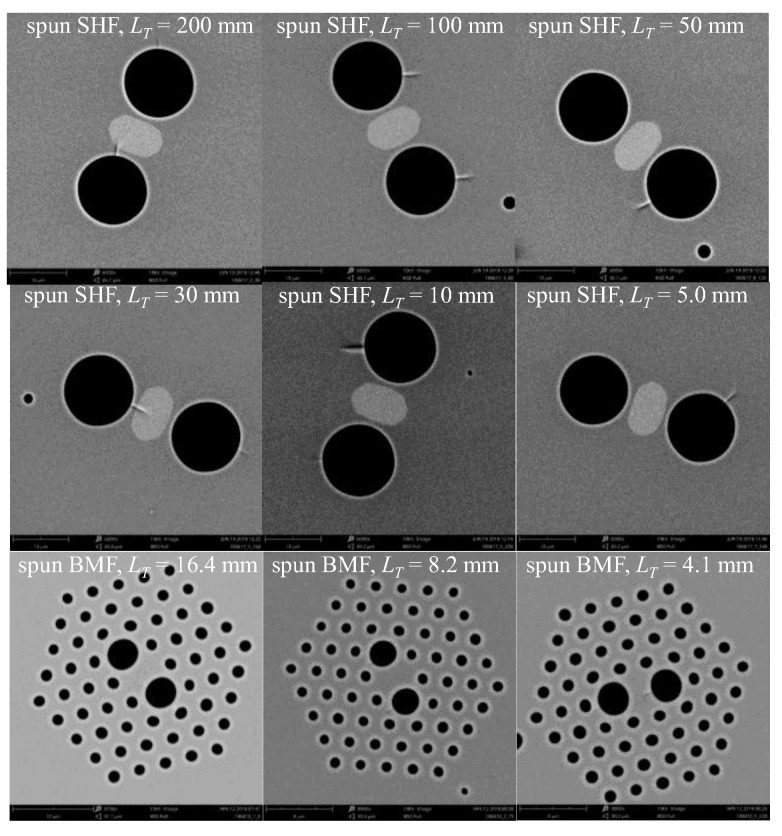
Cross-sections of the spun side-hole fibers (SHFs) and the birefringent microstructured fibers (BMFs) with different spin pitches used in the measurements of torsional sensitivity.

**Figure 5:**
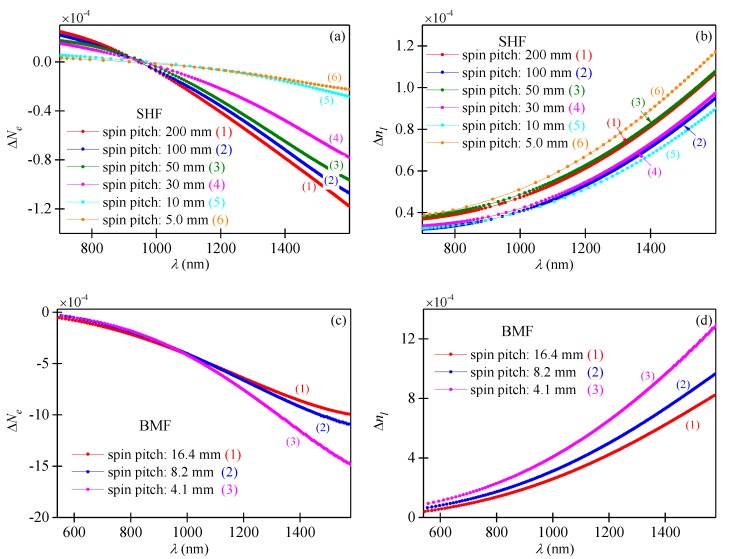
Measured spectral dependencies of group elliptical birefringence Δ*N_e_* and phase linear birefringence Δ*n_l_* in the series of spun side-hole fibers (**a**,**b**) and microstructured birefringence fibers (**c**,**d**).

In the experiments conducted, we examined six side-hole fibers (SHFs) with spin pitches equal to *L_T_* = 200, 100, 50, 30, 10 and 5 mm and three birefringent microstructured fibers (BMFs) with spin pitches equal to *L_T_* = 16.4, 8.2 and 4.1 mm. The cross-sections of all investigated fibers are shown in Figure 4. Using the spectral interferometry method combined with the lateral point-force method [41], we measured the spectral dependence of the phase linear birefringence Δ*n_l_* and the elliptical group birefringence Δ*N_e_* in the investigated fibers (Figure 5). The fibers with different spin pitches were drawn from the same preform rotating with different angular velocities. The measured spectral dependences of Δ*n_l_* varied with the fiber spin pitch, which suggests that the fiber geometry is affected by preform spinning during the drawing process. Our results presented in Figure 5 show that the microstructured fibers having more complex cross-section geometry are particularly susceptible to this effect.

**Figure 6:**
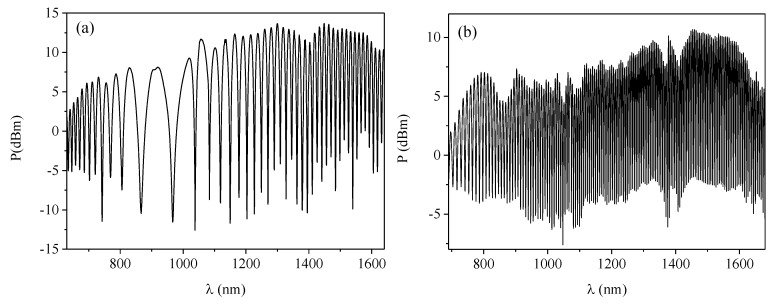
Transmission spectra registered for the side-hole fiber of length *L_0_* = 10 m and spin pitch *L_T_* = 5.0 mm (**a**) and for the birefringent microstructured fiber of length *L*_0_ = 0.35 m and spin pitch *L_T_* = 8.2 mm (**b**).

In the experiments conducted, all pieces of the birefringent microstructured fibers were of the same length *L_0_* = 0.35 m. Longer pieces of the side-hole fibers were necessary to obtain narrow interference fringes, as the group elliptical birefringence Δ*N_e_* of the SHFs was significantly lower than in case of the BMFs (Figure 5). The lengths of the side-hole fibers in the experiments conducted were between 1.5 and 10 m; however, it should be emphasized that neither *L_0_* nor *L_1_* affect the normalized fiber response to torsion as given by Equation (13). The output interference spectra were acquired using Optical Spectrum Analyzer in the range from 650–1600 nm. In Figure 6, we show exemplary output spectra registered for the side-hole fiber and the microstructured birefringent fiber. The sharp peak visible at 1064 nm is related to the residual power of the pump laser used for supercontinuum generation. The characteristic broad interference fringe arising at around 916 nm in the SHF spectrum indicates that the group elliptical birefringence crosses zero at this wavelength. This is not the case for the BMF spectra, as the Δ*N_e_* is negative in these fibers in the whole analyzed spectral range (Figure 5). 

**Figure 7:**
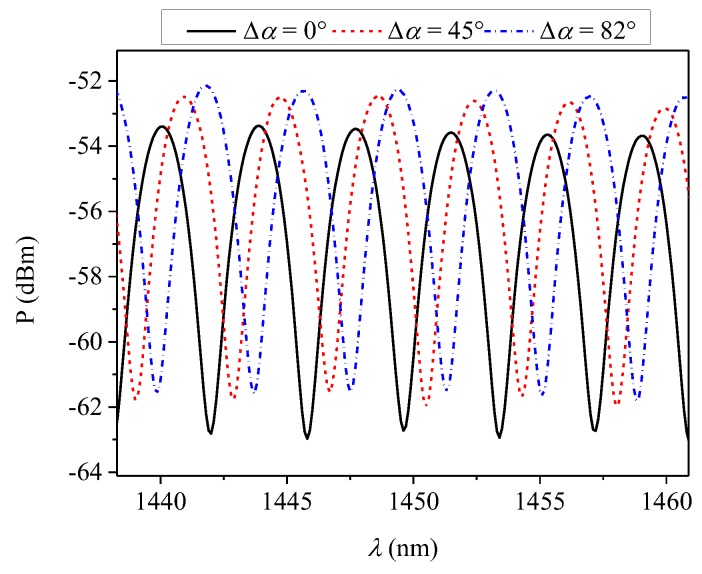
Spectral shift of the interference fringes induced by an additional clockwise twist Δ*α* measured for the side-hole fiber of length *L*_0_ = 10 m (*L*_1_ = 0.25 m) and spin pitch *L_T_* = 30 mm.

**Figure 8:**
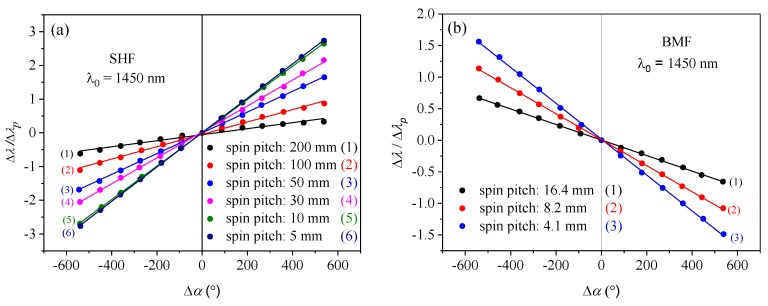
Twist-induced spectral shift of the interference fringe at 1450 nm normalized to fringe width Δ*λ/*Δ*λ_p_* measured for the series of side-hole fibers (**a**) and birefringent microstructured fibers (**b**). The positive sign of Δ*α* corresponds to clockwise twist.

The investigated fibers were subjected to additional twist in the range from −540° (anticlockwise) to +540° (clockwise). According to the analytical results presented in the previous section, the additional twist of the spun highly birefringent fiber caused displacement of the spectral interference fringes in the direction determined by the handedness of the initial fiber twist. In Figure 7, we show the twist-induced displacement of the spectral fringes registered for the SHF of a total length *L_0_* = 10 m (*L_1_* = 0.25 m) and spin pitch *L_T_* = 30 mm. In Figure 8a, the spectral shift is shown for all the examined SHFs measured at the wavelength 1450 nm, while the results obtained for the BMFs are displayed in Figure 8b. The measured wavelength shifts were normalized to the width of the interrogated interference fringe Δ*λ_p_*. By linear fitting of the experimental data, we then determined the normalized sensitivity to torsion *K_α_* for each of the investigated fibers. The obtained results clearly show that *K_α_* increased with the decaying value of the spin pitch *L_T_*. This agrees well with the derived formula for the normalized sensitivity to torsion given by Equation (13). Different signs of the normalized sensitivity *K_α_* for the side-hole fibers and the microstructured fibers was caused by opposite preform spinning directions during the drawing process. The side-hole fibers were spun anticlockwise, and therefore, additional torsion in the clockwise direction resulted in the decrease of the total twist angle *α*. In contrast, the total twist angle of the birefringent microstructured fibers increased when the fiber was subjected to additional clockwise torsion.

**Figure 9:**
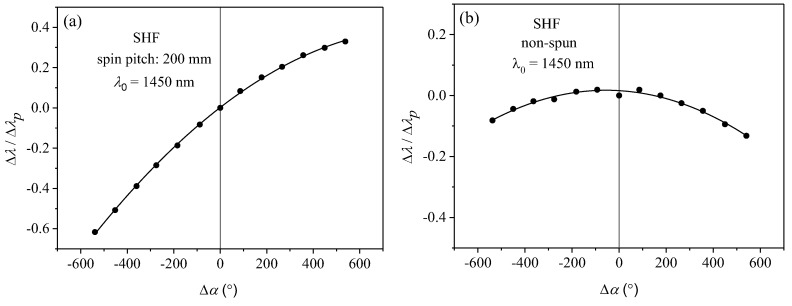
Spectral shift of the interference fringe at 1450 nm measured for the side-hole fiber of length *L*_0_ = 1.5 m (*L*_1_ = 0.25 m) and spin pitch *L_T_* = 200 mm (**a**) and for the non-spun side-hole fiber of length *L_0_* = 1.5 m (*L_1_* = 0.25 m) (**b**).

According to Equation (13), the sensitivity to torsion depends on initial fiber twits; therefore, one may expect a nonlinear relation between fringe shift and applied torsion. This effect, however, is not visible in fibers with initial twist much greater than applied additional torsion, which can be treated as a small perturbation not varying the fiber torsional sensitivity. As is clearly visible in Figure 8, the measured dependencies of the normalized shift of the interference fringes induced by an additional twist match very well the linear trend for most of the investigated fibers, except the side hole fiber with the largest spin pitch *L_T_* = 200 mm. The measured characteristics for this particular fiber are shown separately in Figure 9a. The reason for the visible nonlinear response in this fiber is that its initial twist is small (only 7.5 turns over the total length of *L_0_* = 1.5 m). By applying the additional twist of 1.5 turns in the clockwise or anticlockwise direction, the total number of twists was changed from 6 to 9, which according to Equation (13), resulted in a significant difference in torsional sensitivity.

Additionally, we have measured the response to twist in the non-spun side-hole fiber, which is shown in Figure 9b. According to the analytical results, the response of the non-spun fiber to twist was nonlinear with zero sensitivity at Δ*α* = 0°. Moreover, it is characteristic for a non-spun fiber that the sign of wavelength shift is always negative regardless of the direction of twist Δ*α*.

Thus, we conclude that the high initial twist rate of the spun highly birefringent fibers is particularly advantageous in torsional sensing, as it allows obtaining high torsional sensitivity, as well as a broad range of linear response. The measured torsional sensitivities in the investigated fibers did not differ significantly from the values reported in the literature for other torsion sensors. To make the comparison easier, we have converted the measured normalized sensitivity *K_α_* to the sensitivity expressed in nm/°. For example, as the spectral width of the interference fringe at 1210 nm for the side-hole fiber was equal to Δ*λ_p_* = 21 nm (Figure 6a), the measured normalized sensitivity *K_α_* = 0.2940 1/rad for *L_T_* = 5 mm was equivalent to 0.11 nm/°. This value is of the same order as the sensitivities reported in earlier publications, e.g., 0.19 nm/° [22], 0.08 nm/° [24], 0.07 nm/° [25], and about one order of magnitude lower than the 1 nm/° reported in [23]. 

The experimental values of the normalized sensitivity to torsion *K_α_^exp^* together with linear fitting errors are presented in Table 1 for the SHFs and in Table 2 for the BMFs. The sensitivities *K_α_^exp^* were determined at two wavelengths *λ_1_* = 1210 nm and *λ_2_* = 1450 nm. The response of the weakly spun side-hole fiber presented in Figure 9a was linearly fitted in the small twist range from −180°to +180° where the nonlinear component is negligible. Our experimental results show that the normalized sensitivities measured at two wavelengths were not the same (sensitivity decreased against wavelength), and the average difference reached 5% for the side-hole fibers and 19% for the birefringent microstructured fibers. It is not surprising that the sensitivity was found to be more dispersive for the microstructured fibers, as the birefringence dispersion in these fibers was greater than in the side-hole fibers (Figure 5). 

The theoretical values of the normalized sensitivity *K_α_^t^* were also calculated by using Equation (13) and the birefringence data presented in Figure 5. These theoretical results are gathered in Table 1 and Table 2. The largest mismatch between the measured and calculated values of the normalized torsional sensitivity was found to be 22.6% for the side-hole fiber with the spin pitch equal to 100 mm and 15.7% for the birefringent microstructured fiber with the spin pitch equal to 16.4 mm. 

**Figure 10:**
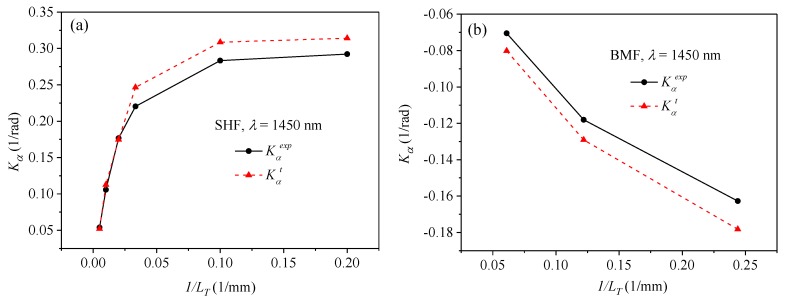
Experimental and theoretical values of the normalized torsional sensitivity for the side-hole fibers (**a**) and the microstructured birefringent fibers (**b**) plotted against the inverse of the spin pitch.

The measured and calculated sensitivities are also presented in Figure 10 for the two families of spun highly birefringent fibers. One should note that only the circular points in the plots represent experimental data, while the straight lines connecting these points were added only for the purpose of improving clarity. It must be emphasized that the torsion experiments (Figure 8) and birefringence measurements (Figure 5) were conducted using the fiber pieces taken from different sections of the spool, which can be a possible reason for the observed differences between measured and calculated sensitivity values. Another reason for the observed discrepancies can be the simplifying assumptions (e.g., neglecting the difference between elastic and nonelastic twist) made to derive the analytical expression for the dependence of torsional sensitivity on initial fiber twist. 

In addition, to confirm the theoretical predictions from Section 2, we have performed temperature sensitivity measurements of spun highly birefringent fibers. First, we have characterized the series of spun side-hole birefringent fibers showing greater *K_T_*. The investigated fibers were stripped of the polymer coating and subjected to temperature changes in the range of 25–110 °C on the length of *L_1_* = 6.0 cm. The linear polarizers at the input and output of the fiber were oriented at 45° with respect to the symmetry axes of the fiber cross-section.

**Figure 11:**
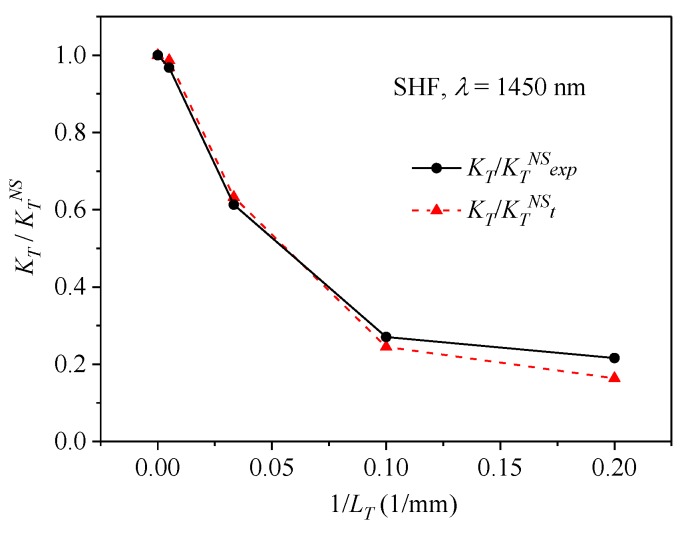
Experimental and theoretical values of the normalized temperature sensitivity ratio $K_{T}/K_{T}^{NS}$ plotted against the inverse of the spin pitch for the side-hole fibers.

In the non-spun fiber, the polarimetric sensitivity normalized to the width of the interference fringe was found to be $K_{T}^{NS}$ = 0.0078 1/K at the wavelength *λ* = 1450 nm (*L*_1_ = 6.0 cm). This value was then used to calculate the ratio $K_{T}/K_{T}^{NS}$ for the spun SHFs. Theoretical values of the $K_{T}/K_{T}^{NS}$ were calculated using Equation (21) and birefringence data presented in Figure 5. In Figure 11, we show both the experimental and the calculated values of the ratio $K_{T}/K_{T}^{NS}$ of the normalized polarimetric sensitivities to temperature. We conclude that there is a good agreement between the experimental and theoretical values, which supports our analytical findings presented in Section 2. In particular, our results show that the side-hole fiber with the smallest spin pitch exhibited the lowest normalized sensitivity to temperature while its sensitivity to torsion was the highest. 

We have also investigated the temperature sensitivity of the spun highly birefringent microstructured fibers in the same experimental setup. Because the *K_T_* in non-spun birefringent microstructured fibers was about two orders of magnitude lower than in the side-hole fibers [43], the temperature-induced displacement of interference fringes was below the measurement resolution and therefore not detectable. This observation is in agreement with the experimental results from [45] showing no change in group birefringence within the experimental error range observed for the spun highly birefringent microstructured fibers in the temperature range between −25 and 100 °C. 

## 4. Conclusions

In this paper, we have studied the polarimetric sensitivity to torsion and temperature in spun highly birefringent fibers. The parameter of interest was the measurand-induced displacement of spectral interference fringes arising in the output spectrum because of the interference of elliptically-polarized modes. We have examined two series of spun highly birefringent fibers, i.e., the side-hole fibers and the birefringent microstructured fibers with differing birefringence dispersion and spin pitches. The two series of fibers were spun in opposite directions, which resulted in the opposite sign of torsional sensitivity. Our experimental results show that the absolute value of the normalized torsional sensitivity grows with decreasing spin pitch, while at the same time, the range of the linear response to torsion broadens. In contrast, the sensitivity to temperature asymptotically decreases to zero with increasing twist rate. Thus, it is advantageous to use the fibers with a high initial spin rate for torsion measurements. 

In order to better understand the experimental results, we have derived analytical relations linking the value of torsional sensitivity with fiber intrinsic linear birefringence and spin pitch. By using the derived formula, we have calculated the theoretical values of the normalized torsional sensitivity in the examined fibers. The experimental and theoretical values match qualitatively with the greatest difference reaching 22.6%. The theoretical analysis implies that the normalized torsional sensitivity tends asymptotically to the value of 1/π rad^−1^ with increasing initial fiber spin. 

Moreover, we have also analyzed theoretically the sensitivity to temperature, which in contrast to torsional sensitivity decays with increasing fiber spin. These predictions were confirmed by measurements of the temperature sensitivity in spun and non-spun side-hole fibers. For the 6 cm-long piece of the spun side-hole fiber with *L_T_* = 5 mm, the sensitivity to torsion and temperature measured at *λ* = 1450 nm were equal, respectively *K_α_ =* 0.29 rad^−^^1^ and *K_T_ =* 0.0016 K^−^^1^, which results in the cross-sensitivity coefficient between torsion (expressed in radians or arc degrees) and temperature equal to 0.0055 rad/K (0.32 °/K). As the polarimetric sensitivity to temperature in birefringent microstructured fibers is about two orders of magnitude lower than in side-hole fibers, the expected value of the cross-sensitivity to temperature in microstructured fibers was only 3 × 10^−3^ °/K, which allows for direct torsion measurements without the need for any temperature compensation. Thus, we conclude that another advantage of using the fibers with short spin pitches for torsion sensing is their low cross-sensitivity to temperature. 

## Figures and Tables

**Table 1:** Experimental and theoretical values of the normalized sensitivity to torsion in the spun side-hole fibers at wavelengths *λ*_1_ = 1210 nm and *λ*_2_ = 1450 nm.

	***λ*_1_ = 1210 nm**				
***L_T_* (mm)**	***L_L_*** **(mm)**	***K_α_^exp^*** **(rad^−1^)**	**Δ*K_α_^exp^*** **(rad^−1^)**	***K_α_^t^*** **(rad^−1^)**	**(*K_α_^exp^* − *K_α_^t^*)/*K_α_^exp^*** **(%)**
200	19.5	0.0596	2.3 × 10^−3^	0.0609	−2.18
100	22.5	0.106	2 × 10^−3^	0.1301	−22.6
50	19.1	0.1948	1.2 × 10^−3^	0.1929	0.98
30	21.9	0.2323	1.5 × 10^−3^	0.2626	−13.0
10	23.3	0.2919	4.3 × 10^−3^	0.3112	−6.6
5.0	17.7	0.2940	3.1 × 10^−3^	0.3152	−7.21
	***λ*_2_ = 1450 nm**				
***L_T_* (mm)**	***L_L_*** **(mm)**	***K_α_^exp^*** **(rad^−1^)**	**Δ*K_α_^exp^*** **(rad^−1^)**	***K_α_^t^*** **(rad^−1^)**	**(*K_α_^exp^*** **− *K_α_^t^*)/*K_α_^exp^*** **(%)**
200	16.6	0.0538	2.1 × 10^−3^	0.0521	3.16
100	18.9	0.1058	3.1 × 10^−3^	0.1126	−6.43
50	16.4	0.1766	0.9 × 10^−3^	0.1748	1.02
30	18.4	0.2203	1.5 × 10^−3^	0.2465	−11.9
10	19.8	0.2833	0.6 × 10^−3^	0.3086	−8.9
5.0	15.1	0.2922	0.8 × 10^−3^	0.3140	−7.46

**Table 2:** Experimental and theoretical values of the normalized sensitivity to torsion in the spun birefringent microstructured fibers at wavelengths *λ*_1_ = 1210 nm and *λ*_2_ = 1450 nm.

	***λ*_1_ = 1210 nm**				
***L_T_* (mm)**	***L_L_*** **(mm)**	***K_α_^exp^*** **(rad^−1^)**	**Δ*K_α_^exp^*** **(rad^−1^)**	***K_α_^t^*** **(rad^−1^)**	**(*K_α_^exp^* − *K_α_^t^*)/*K_α_^exp^*** **(%)**
16.4	2.8	−0.0894	4.6 × 10^−4^	−0.1034	−15.7
8.2	2.4	−0.1473	5.8 × 10^−4^	−0.1593	−8.16
4.1	1.8	−0.1948	1.1 × 10^−3^	−0.2109	−8.26
	***λ*_2_ = 1450 nm**				
***L_T_* (mm)**	***L_L_*** **(mm)**	***K_α_^exp^*** **(rad^−1^)**	**Δ*K_α_^exp^*** **(rad^−1^)**	***K_α_^t^*** **(rad^−1^)**	**(*K_α_^exp^* − *K_α_^t^*)/*K_α_^exp^*** **(%)**
16.4	2.1	−0.07047	5.2 × 10^−4^	−0.08015	−13.7
8.2	1.8	−0.1180	5.8 × 10^−4^	−0.1291	−9.39
4.1	1.4	−0.1627	9.2 × 10^−4^	−0.1782	−9.53
